# The rural health initiative: Bridging gaps in healthcare access and grant-funded research in central Idaho

**DOI:** 10.1017/cts.2024.669

**Published:** 2024-12-12

**Authors:** Monica Zigman Suchsland, Dillon van Rensburg, Kelly McGrath, Cody Wilkinson, Kimberly Johnson, Ashley Steinbruecker, James Probus, Allison Cole

**Affiliations:** 1 University of Washington, Department of Family Medicine, Seattle, WA, USA; 2 University of Washington, Institute of Translational Health Sciences, Seattle, WA, USA; 3 St Mary’s Health and Clearwater Valley Health Central, ID, USA

**Keywords:** Community engagement, rural health, healthcare access, social determinants of health, priority setting

## Abstract

**Introduction:**

Translational science rarely addresses the needs of rural communities, perpetuating health inequities. Furthermore, policy and resource allocation reflect this dynamic. Through a partnership between a rural community and a community engagement program, the Rural Health Initiative (RHI) was developed with the goal of building capacity for community-driven translational research in rural settings.

**Methods:**

We describe the process of forming the RHI and selection of a community health priority to motivate the translational research agenda in this particular rural setting. We used a mixed methods approach utilizing literature review, community survey data, and qualitative evaluation of community meeting discussions. Consensus on a final health priority was built through voting and comparison of voting responses across the three RHI counties through Fisher’s Exact test.

**Results:**

Four priority topics were identified through literature search, community needs assessment, state/national trend data, and community experts. Priority ranking from a community forum and survey selected the final health priority topic. Healthcare access was selected by all three counties in the RHI community as the most critical health priority to address.

**Conclusions:**

This program highlights the importance of and methods for community involvement in directing the research conducted in their community. Additionally, through this project, guidance was developed to define the role of community engagement programs supporting work led by communities.

## Introduction

Inequities between rural and urban communities have been a steadfast issue. The National Institutes of Health (NIH) has long recognized that living in a rural area is associated with shorter life expectancy and higher prevalence of disease compared to the United States (US) population overall [1]. Rural areas in the US experience a greater mortality rate and lower rates of healthy lifestyle behaviors that reduce the incidence of chronic disease compared to urban areas in the US [2,3]. Rural communities also experience limited services and treatment availability to address the higher burden of disease compared to urban areas [4,5]. While national organizations, such as the NIH and the Centers for Disease Control and Prevention, have called upon health researchers to specifically evaluate the inequities created by social determinants of health such as geography of residence (rural/urban), most published translational research does not focus on rural populations or settings [1,6,7]. The lack of translational research investment, such as limited recruitment in rural populations or untailored health interventions, perpetuates health inequities for rural populations, leaving unmet health needs in these communities.

Research partnerships between academic clinical researchers and rural health care practitioners and their patients have been advocated as a potential solution to improving adoption of translational research in rural settings [7,8]. Programs primed to foster such partnerships are Community Engagement (CE) programs often funded through Clinical and Translational Science Awards (CTSA). CTSA programs provide the infrastructure for CE, workforce development, research training, and support of translational research with the overall goal to bridge the gap between health research, equity, and practice [9]. CE programs facilitate research that addresses community priorities and reduces barriers for communities to participate in academic or clinical research [8]. The Institute of Translational Health Sciences (ITHS), the University of Washington’s (UW) CTSA program, is uniquely positioned to improve rural health equity through the rural community relationships it has maintained and the translational health resources it can dispatch through the CE program to rural communities in the region. These specific attributes align closely with a proposed rural implementation model developed to reduce rural health inequities by 1) leveraging rural communities’ strengths by investing in relationships as an implementation asset; 2) funding and building creative individual and organizational capacity in rural communities; and 3) partnering with rural communities to translate research-based programs and practices to the rural context [10]. Community-engaged rural health research can therefore improve health outcomes specific to rural communities through selected health priorities and community-informed interventions.

The UW ITHS CE program partners with rural communities across the five-state WWAMI (Washington, Wyoming, Alaska, Montana, and Idaho) region on clinical and translational research. The Rural Health Initiative (RHI), one such partnership between a community comprised of three counties in north central Idaho and UW ITHS CE, was developed to provide the local community with a unique opportunity to lead a research effort. The goal of the RHI is to develop a rural community health research program to address a critical health priority selected by the rural community. The RHI was developed to meet the following three objectives: 1) Promote research that improves health outcomes related to a critical priority selected by the community; 2) Build capacity of rural communities and investigators to collaborate in clinical and translational science; and 3) Develop and disseminate innovative and effective approaches to support rural community and translational research partnerships. In this manuscript, we outline the methods and outcomes for engaging with community partners to identify and prioritize potential rural health challenges as part of a rural health initiative. We will describe this unique partnership by outlining the context and goals for the RHI, methods for the health priority selection and comparison across the region, and share recommendations for best practices for research engagement with rural community partners.

## Methods

### Setting

**Figure 1.**
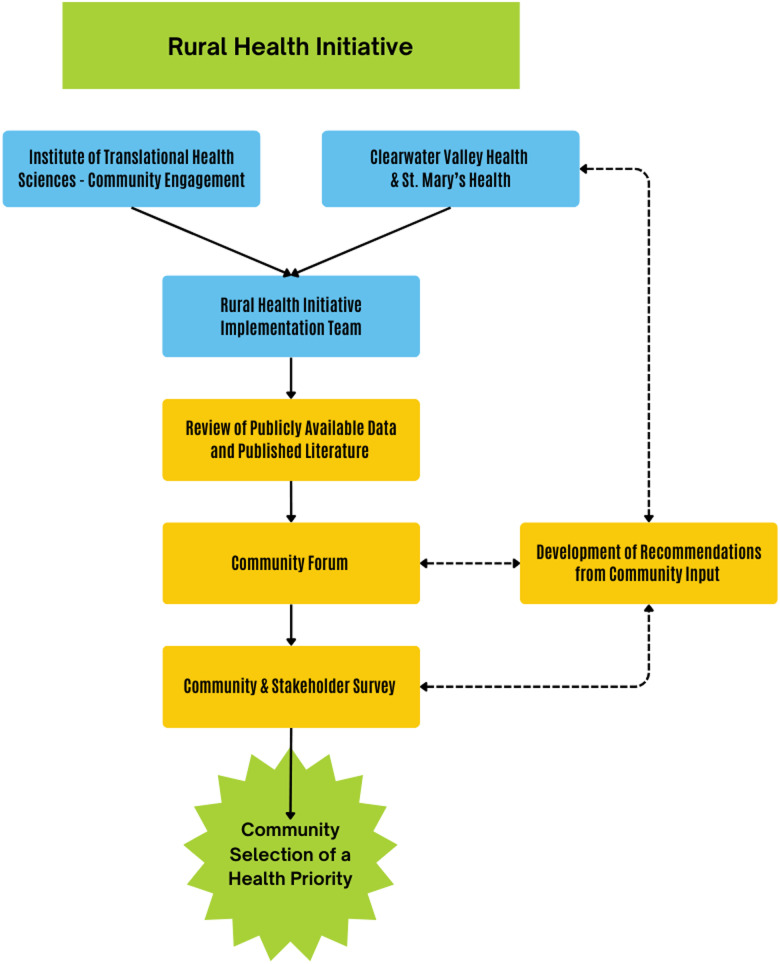
The rural health initiative health priority selection process.

The Rural Health Initiative includes two partners: the ITHS CE program, a university-supported program, and the St Mary’s Health and Clearwater Valley Health System (SMH-CVH), the community collaborator. The SMH-CVH system serves Clearwater, Lewis, and Idaho counties, all parts of north central Idaho. The health system has two critical access hospitals and eight primary care clinics. It serves a population of 29,000 residents over a land area similar to the size of Massachusetts. According to the 2020 US Census, 3% of residents are American Indian and Alaska Native, 4% are Hispanic or Latino, 1% other races, 5% are two or more races, and 86% are White. SMH-CVH conducts regular community health needs assessments to direct health services and tailor healthcare strategies to the specific health needs of the community. The RHI leadership, SMH-CVH (KM) and ITHS CE (AC), designed the concept of this project together, a community and academic partnership that centers the community at the heart of a research program. They assembled a Rural Health Implementation Team consisting of experts in population health and needs assessment at SMH-CVH as well as experts in community engagement and research management at ITHS CE. The Implementation Team met monthly (or more frequently when needed), beginning in June 2022. The Implementation Team designed the goals of the RHI and developed the process for the selection of the community health priority (Figure 1). The Implementation Team relied heavily on the experiences and community knowledge of the SMH-CVH team members to direct the project decisions. RHI activities centered on the communities in the north central Idaho region leveraging community partnerships and relationships through the SMH-CVH team, while ITHS CE team located in Seattle, Washington provided support, co-led the in-person community forum, and coordinated academic partnerships with the RHI. Funding supported both the community partners, SMH-CVH team, and the academic partners, ITHS CE, equally for participation in this project. Self-determination of whether an activity is human subjects research by Common Rule, allowed by the University of Washington Human Subjects Division, was conducted. The research team determined this project did not meet the definition of human subjects research.

## Health priority selection

### Review of publicly available data and published literature

A review of publicly available data sources reporting disease, death, or healthy lifestyle behaviors for the state of Idaho or more specifically Clearwater, Idaho, and Lewis counties was conducted in the summer of 2022. Overview data such as mortality rate for the leading causes of death and diseases with high prevalence in this region compared to the general US population were extracted and summarized by the ITHS CE members of the RHI Implementation Team. Summary data were reviewed by the entire RHI Implementation Team. Of the data presented, the eight health topics that had the highest prevalence and were determined locally by the SMH-CVH members of the RHI Implementation Team to be unmet health needs in the community were further investigated by the ITHS CE members of the RHI Implementation Team for more information in the scientific literature and publicly available data. Again, data was summarized by topic and the list was narrowed down in a similar process by the entire RHI Implementation Team to a final list of four potential health priority topics for north central Idaho. The four potential health priority topics were then presented at a community forum priority-setting meeting.

### Community forum

In October 2022, the Rural Health Initiative Implementation team convened in Kamiah, Idaho to conduct a community forum. Kamiah, Idaho is a small town situated along the Clearwater River in the north central region of the state. The town has a population of approximately 1,300 people and is given a Rural Urban Continuum Code of 8. Kamiah, Idaho is also a central location within the counties where the RHI plans to implement the health priority project. The purpose of the forum was to introduce the Rural Health Initiative to the community and select a community health priority in north central Idaho. The population health and needs assessment experts at SMH-CVH leveraged their community contacts to invite community leaders from the health system, public health district, and local community-based organizations to participate in the community forum. Community members were contacted through email and phone, and community members from the health system, school boards, food banks, and local government officials were invited. Compensation for time was not provided but a meal was served to attendees.

The Implementation Team presented background data specific to the four health topics identified during the initial RHI planning. Following each individual topic presentation, the RHI team facilitated small group discussions where community members highlighted solutions and barriers to addressing these health topics. At the conclusion of the forum, each community member voted to identify the health topic they deemed most important to address. Additionally, attendees completed an evaluation of the event.

### Community survey & stakeholder survey

A community survey was developed to include a broader range of community perspectives outside of those in attendance at the community forum. The community survey was a series of ten questions, with four questions asking about the level of importance of each of the four health topics presented at the community forum (e.g., In your opinion, how important is it to address obesity / healthy lifestyle in your community?) provided on a Likert scale from “not important at all” to “very important,” a vote for the most important topic, and an open-ended question to provide further comment on the topic. Four questions on the survey were demographic and asked about the participant’s age, gender, race, and county. The survey was developed via REDCap and distributed via hyperlink to a web survey to other community partners in the region through an SMH-CVH listserv. The listserv comprised community leaders and members from around north central Idaho who had previously worked with the hospital in some capacity previously, such as participants from the community health needs assessment routinely completed by SMH-CVH. The listserv encompassed both healthcare professionals and those outside the healthcare system (e.g., foodbank, school district). The survey was open for 3 weeks from January 12, 2023 – February 3, 2023.

After the completion of the community forum and survey, an in-depth Stakeholder survey was created. The goal was to gather information from community stakeholders on what aspect(s) of the selected health priority were most important and most feasible to solve in their community. Participants were asked to rate the level of importance for different health topics, describe barriers to healthcare access, and provide open-ended comments after each question. The survey was open for one month from March 13, 2023 to April 18, 2023.

## Analysis

### Quantitative data

At the close of the Community Forum, each participant was asked to rank the four health priorities in order of importance (1-most important to 4-least important). Total points were calculated and the health priorities were ordered from most important to least important. Summary statistics were calculated for the community survey. A Fisher’s exact test for independence was run to test the null hypothesis that the most important health topic to address in the community was the same across the three counties that make up the RHI (Clearwater, Lewis, and Idaho counties). Statistical analysis was conducted in RStudio (Version 2023.06.0.421, Mountain Hydrangea Release) and significance was set at *p* = 0.05 [11].

### Qualitative data

Detailed notes from the community forum assisted in identifying the RHI key health priority. Notes from the three notetakers were compiled into a single document, uploaded to Dedoose qualitative analysis software (Dedoose Version 9.0.107), and organized by potential health priority topics for analysis [12]. Additionally, open-ended responses to both the community and Stakeholder surveys were organized and uploaded to Dedoose for coding. An initial coding template was created, where each health priority topic was a parent code, with “facilitators, barriers, thoughts on the topic, and additional information/miscellaneous” sub-codes under each health topic.

Two members of the research team (DV and MZS) independently reviewed the responses to become familiar with the content. Inductive coding was then initiated where excerpts were labeled based on their most noticeable and important characteristics according to the coding template. The coders (DV and MZS) met to review and reconcile coding discrepancies. DV consolidated excerpts into groups based on their commonalities and the central ideas that underlie them. Thematic analysis was used to identify themes for major barriers and facilitators to addressing health priorities. The full analytic team (AC, DV, and MZS) conducted a thorough review of the excerpts and deductively identified general theme groups. MZS reviewed the excerpts in the theme group and developed thematic definitions. The full analytic team verified that these defined themes exemplified the coded segments of data. Finally, the analytic team assessed how these themes connected with the overarching goals of the Rural Health Initiative.

Upon conclusion of the literature review, community forum and the closure of both the community survey and stakeholder survey, the Implementation Team would review event evaluation or survey results. The team would discuss important findings and what worked well for each method. Key takeaways from these discussions were developed and iteratively refined into recommendations for rural community engagement.

## Results

### Publicly available data review

Regional and national morbidity and mortality data was collected from 13 publicly available data sources (Supplement: S.1 Publicly Available Data Sources). The eight potential health priority topics selected from the collated summaries of these data reports included: alcohol use, cancer screening, skin cancer, obesity and healthy lifestyle, health workforce and healthcare access, deaths of despair, lung cancer, and vaping/marijuana use. After additional data search, a final four potential health priority topics were selected for review at the Community Forum: cancer screening, obesity and health lifestyle, health workforce and healthcare access, and deaths of despair.

## Survey results

### Community forum

There were 12 community members in attendance at the community forum meeting. Majority were female (*N* = 8), over the age of 40 (*N* = 10), and all identified as white (Table 1). Half the attendees worked in healthcare or hospital settings, two attendees were from community-based organizations, one attendee was from a government agency, and 3 declined to identify where they worked. On a vote for the most important health priority presented at the community forum 54% selected healthcare access as their number one health priority, 23% selected obesity & healthy lifestyle, 15% selected deaths of despair, and 8% selected cancer screening.

**Table 1.** Voting results from Kamiah, Idaho community forum

Total N – 12		
Demographics	Frequency	%
Organization		
Community-based	2	17
Government	1	8
Hospital and Healthcare	6	50
Prefer not to answer	3	25
Age		
18–39	2	17
40–64	10	83
Gender		
Male	3	25
Female	8	67
Prefer not to answer	1	8
Race		
White	12	100
Health Priorities Ranked as Number One [1]	Category	%
1^st^ Place	Healthcare Access	54
2^nd^ Place	Deaths of Despair	15
3^rd^ Place	Cancer Screenings	8
4^th^ Place	Obesity & Healthy Lifestyle	23

*Note*: 1) One participant completed the health priority ranking but did not complete demographics, giving a total of 13 participants.

Evaluation from the event had overall positive ratings. All attendees selected responses “agree” or “strongly agree” to the following: *A respectful atmosphere was created; The understanding of differing viewpoints was encouraged; A sense of partnership among community members and the RHI team was present; The RHI team understood and supported the role of community members; The RHI team was responsive to input from community members; There was clear communication about how community member input will be incorporated into decisions*. There were two questions where one community member selected “disagree” (all others selected “agree” or “strongly agree”): *The stated goals of community involvement were achieved; Community members were given adequate opportunity to share their perspectives*. All attendees agreed that at least one of the health topics presented at the Forum was important to the community and addressing at least one would have a positive impact on their community. Additionally, they all agreed that the community partnership with the RHI can address at least one of the health priorities presented. There was more of a mix in responses when considering barriers in the community, 42% agreed that there were too many barriers in the community to address any of the health priorities discussed.

### Community survey

There were 139 participants who completed the community survey. Most participants (102, 73%) identified themselves between the age groups of 35–64 (Table 2). There were 120 (86%) participants who identified themselves as female while 14 (10%) as male. For race, six (4%) participants identified as Asian, five (3%) as Hispanic/Latino, five (3%) as Native American, one (0.7%) as Pacific Islander, and 117 (84%) as White. Regarding counties central to the Rural Health Initiative, 50 (36%) participants resided in Clearwater, 60 (43%) in Idaho, and 16 (12%) in Lewis counties. There were eight (6%) participants who lived on the Nez Perce reservation.

**Table 2.** Demographics and health priorities from a survey of individuals in central Idaho

Total N – 139		
Demographics	Frequency	%
What is your age group?		
18–24	6	4.3
25–34	16	11.6
35–44	40	29
45–54	25	18.1
55–64	37	26.8
65–74	11	8
75–84	2	1.4
Prefer not to answer	1	0.7
What is your gender?		
Female	120	88.2
Male	14	10.3
Prefer not to answer	2	1.5
What’s your race?		
Asian	6	4.4
Hispanic/Latino	5	3.7
Native American	5	3.7
Pacific Islander	1	0.7
White	117	86.7
Prefer not to answer	8	5.9
In what county do you live?		
Clearwater	50	36
Idaho	60	43.2
Lewis	16	11.5
Nez Perce	8	5.76
Prefer not to answer	3	2.16

Health Priorities	*Not Important* *at all*	*Somewhat Important*	*Important*	*Very* *Important*
Obesity & Healthy Lifestyle: In your opinion, how important is it to address obesity / healthy lifestyle in your community?	2 (1.4%)	9 (6.5%)	48 (34.8%)	79 (57.2%)
Deaths of Despair: In your opinion, how important is it to address deaths of despair in your community?	1 (0.7%)	8 (5.8%)	42 (30.4%)	87 (63.0%)
Cancer Screenings: In your opinion, how important is it to address cancer screening in your community?	1 (0.7%)	6 (4.3%)	51 (36.7%)	79 (56.8%)
Healthcare Access: In your opinion, how important is it to address health care access in your community?	1 (0.7%)	6 (4.3%)	27 (19.4%)	104 (74.8%)
	*Obesity & Healthy Lifestyle*	*Deaths of Despair*	*Cancer Screenings*	*Healthcare Access*
Out of the four health topics, which topic do you believe is the single most important to address in your community right now?	36 (26.3%)	29 (21.2%)	12 (8.8%)	60 (43.8%)

*Note*: One participant was not included in the total due to them living outside of central Idaho.

Obesity and healthy lifestyle, deaths of despair, cancer screenings, and healthcare access were all generally viewed as important to address in the community, with most votes residing in the “important” and “very important” range of the Likert scale. However, 104 participants (75%) selected “very important” when it came to addressing healthcare access in their community. There were 87 participants (63%) who indicated deaths of despair as “very important” while 79 participants (57%) selected cancer screenings and obesity and healthy lifestyle as “very important,” respectively.

For community members residing in Clearwater, Idaho, and Lewis counties, there was no difference between these counties in response to their selection of the most important health topic between healthy lifestyle, deaths of despair, cancer screening, and healthcare access. On a Fisher’s exact test for independence, we fail to reject the null hypothesis (*p* = 0.74). Of the topics listed, healthcare access was selected the most frequently in all three counties (42% Clearwater, 45% Idaho, 43.8% Lewis county).

## Qualitative results

The data aligned with two broad thematic categories, barriers, and solutions to the selected health priority. Barriers and solutions emerged across all 4 health priorities discussed: access to care, deaths of despair, cancer screening, and obesity/healthy lifestyle; additionally, across all three data sources, the community forum discussion, community survey, and stakeholder survey. Four themes were identified under the barriers category and five themes were identified in the solutions category. Each is discussed below with exemplary quotes.

### Barriers

Geography of the region was described as a consistent barrier for many of the health topics. Distance from essential services and other community members or lack of infrastructure present burdens that limit the ability of the community to access services or connect with one another. One participant described the geography barrier, “*I see access is a big issue, to get the services needed, will have to travel a minimum of an hour, but many times, it is 3–4 hours*.” Another described distance of resources in relation to gaps in services given the large land area and small population, “*Transportation is a major need in this part of Idaho. We have small communities who are not served at all by Medicare/Medicaid transportation providers. We also have many patients who go to [nearest city] for cancer treatment and have to rely on family and some do not have family and exhaust community members who try to help.*” Other infrastructure needs were shared such as internet bandwidth, food accessibility, or safe places to walk (no sidewalk, lighting, or established walking trails).

Another barrier identified by the community was the theme of economics. This included limitations in insurance coverage and high out-of-pocket costs along with lower socioeconomic status of some residents in the region. It was described here, “*Cost and dealing with insurance keeps a lot of people out; people put it off. Care can be expensive and even if it’s covered, getting insurance coverage and tracking coverage takes a lot of time*.” The healthcare workforce shortage also presented barriers, hindering the capacity of the health system to provide services. This theme is summarized by the following quote, “*As our community grows, our providers and healthcare staff face extreme burnout. All while not feeling fairly compensated (especially in terms of benefits) for the jobs that they do.*” The last barrier identified was the local culture of the region. The impact of stigma and local beliefs prevents some from accessing healthcare, “*Old farm mentality- they just dig their heels in. “I do not doctor.” Hard to get through to some folks, we need better outreach.*” Adding to local culture is the closeness of the community which affects how people access healthcare. “*In small communities, it’s harder to maintain anonymity and deal with embarrassment, social comparison.*”

### Solutions

There were five potential solutions identified in the thematic analysis. These included education, development of new or expansion of existing services, cost reduction, supporting behavioral changes, and developing infrastructure. Health education for health topics that impact the community was mentioned as an area for addressing health challenges in the community; “*Community Health Workers, home health can assist in providing education and increasing access, especially for patients who are very difficult to reach.*” Additionally, education was described as a method to tackle some of the complex health challenges facing the community, “*We have had a rise in Fentanyl related deaths in our surrounding communities. Maybe a class on how to administer Narcan as well as community education on Fentanyl would be helpful.*” Similar to education, developing new services or expanding existing services was seen as a way to improve healthcare access. Increasing funding or capacity to improve the size of the healthcare workforce, reduce wait times, and add services was described. Novel services included, “*School-based telemedicine delivery could help overwhelmed counselors and meet the needs of children*,” or, “*Community health workers- sometimes people do not need a doctor, and other support personnel might be more readily available.*”

The next potential solution addresses the economic barriers to health and healthcare access specifically. Cost reduction, meaning providing services for free or at discounted rates and improving insurance coverage was identified as a priority. This was described simply in the data, “*Affordable healthcare access would be beneficial for a patient that has a high deductible.*” Additionally, another potential solution is programs and policies that target behavior change. There were programs or policies mentioned that target unhealthy behaviors or encourage healthy behaviors. For example, “*Community-based fitness plans like the Mayor’s Challenge could be useful- create routine group walking events or basic fitness classes that are free to attend and leverage social connections*.” One additional solution was improving the overall infrastructure of the region. This theme described building systems that overcome the obstacles that arise from providing services to large land areas traditionally under-resourced. Two such systems that were specifically suggested include telehealth expansion and community health worker programs. “*Telehealth could be useful here since the in-person staff are already swamped.*” These solutions were explained as ways to address unhealthy behaviors or improve access to care.

## Discussion

The initial phase of the RHI outlines the development of a novel CTSA/community partnership to design a community-led research program. The community/academic partnership bridges unique expertise in both translational science and community engagement. Through this partnership, the RHI Implementation Team successfully leveraged publicly available data and the scientific literature to narrow down to a select number of health topics that disproportionally impacted north central Idaho more than the rest of the state or country. We employed several methods to explicitly have the community select the health priority they wanted to be addressed. This included a community forum to provide space for in-depth discussions with community leaders, a broader community survey to reach more voices in the community, and a stakeholder survey to understand specifics about the health priority. The community selected healthcare access as the top health priority for north central Idaho. Barriers that amplify the lack of access to healthcare include geography, economics, workforce, and local culture. Potential solutions that may improve healthcare access highlighted by the community are education, development of new or expansion of existing services, cost reduction, behavioral changes, and infrastructure.

Our experience demonstrates the importance of early and sustained partnership with community leaders. Though rural health disparities have long been recognized, engagement with rural communities to design and implement research programs to address these disparities has been limited [13,14]. Among research projects that include engagement with rural community partners, co-leadership with rural partners remains exceedingly rare [15]. To address this gap, we intentionally designed the RHI Implementation Team to be co-led by a CTSA faculty (AC) and a local medical director (KM) and includes members of the CTSA and local health system. Through this model of co-leadership and collaborative implementation planning, we were able to design an approach that engaged community leaders and community members in selecting a critical health priority on which the RHI can focus.

Building successful community-academic partnerships requires investment of resources and time [16]. Each partner has a unique role in the partnership development and implementation [17]. In our approach, the academic team time was supported by the CTSA program and community members were representatives of the local health system, which partnered in the project. The academic partners brought experience and resources in scientific methods and program management, while the community leaders brought deep expertise in community structure, engagement, and local priorities. These synergistic areas of expertise and resources supported the partnership and sustained it through the engagement and planning process [18].

This manuscript lays out the foundational steps for a community-led research initiative. However, we recognize there are several limitations to the process we have outlined here. First, to select initial potential health priorities, we were limited by the national and local data that is publicly available. There may be gaps or limitations to data collected on national surveys. Additionally, data lag real-time trends, so some information may have been out-of-date. To fill in some missing information the team relied on the local community needs assessment results conducted in 2019. Thus, we may not have fully considered key health priorities not evident in the data to which we had access. Another limitation to this initiative may have been the representation and voices heard at the community forum. As with any group discussion hearing from all the voices can be a challenge so we designed the format for small group discussion and share back to hear from everyone. We relied on the SMH-CVH team to invite representative community members. Last, to ensure the final health priority selected at the community forum was in fact a priority for the entire community, we dispatched the community survey to over 100 community members, which was not part of the original priority selection methods.

## Conclusions

It is pivotal to identify best practices that have been learned or reinforced during the beginning stages of the RHI. We believe these recommendations foster meaningful and effective collaboration between academic institutions and rural community partners, which include:

1. *Encourage Academic and Community Partners to Share their Recommendations and Perspectives:* There is a mutual benefit for academic institutions and rural community partners to feel comfortable and confident in sharing their recommendations and perspectives on research. Academic institutions have valued resources and guidance to carry studies to fruition, while rural community partners offer deeper, more accurate insights into local needs and solutions. This exchange builds trust, leverages existing programs, identifies care gaps, and mitigates cognitive biases to nurture a more holistic view of the challenges at hand.
2. *Implement Community-Driven Processes:* Incorporating community-driven processes into rural research initiatives is vital. Processes can include co-principal investigators, community advisory boards, and participatory action research, where community partners actively shape research design and execution. These methods support rural research that is beneficial for everyone involved. In our case, the academic institution team would likely have selected a different health priority that was important for their institutional goals rather than select a health priority that meets the community partner’s biggest needs. By utilizing community-driven processes, our team collectively came to a health priority in both a synergistic and optimal way that would not have been evident working in isolation.
3. *Build Appropriate Partnerships:* Be cognizant when starting new partnerships for rural research by ensuring all individuals involved have a shared mission and purpose, which is essential for effective collaboration. For academic institutions, partner with community organizations who recognize the community’s needs and culture, have connections to both community leaders and members and are motivated to collaborate. For community partners, collaborate with academic institutions who genuinely appreciate and comprehend the distinct characteristics and complexities of the community. These institutions should also demonstrate their trustworthiness and their willingness to engage in the research process directly within the community, ensuring a more personal and in-depth understanding of community context.
4. *Aim for Inclusive and Adaptable Engagement:* Give ample time and space for community voices to be heard through multiple platforms. From the RHI, we spent several months collating different community perspectives through surveys and in-person community forums. These approaches provided deeper context for health barriers and facilitators and made us confident in our selection of healthcare access as the top health priority. It is also important to recognize and respect divergent priorities from those of the academic institution. It is important for all team members to be adaptable and flexible in research methods and approaches based on community feedback.
5. *Sustain a Long-term Commitment:* Establish a long-term commitment to the community. For RHI, the research team had been collaborating with the community partnering organization for several years. This continued partnership from previous research studies made for a trusting relationship that also increased our confidence in taking on larger, more complex projects, such as starting a new program.

The initial phase of the RHI has provided valuable insights into fostering collaborative relationships between academic institutions and rural communities. The lessons learned point towards the necessity of a balanced approach that respects community dynamics and prioritizes shared objectives. Emphasizing community-driven processes and inclusive engagement, this experience suggests a pathway for similar initiatives, highlighting the importance of adaptability and mutual understanding. Next steps for the RHI include development of a community-led project to address healthcare access, mental healthcare access to start, and building collaborations with scientific investigators working to improve healthcare access. The final step for RHI will be evaluation of the program including number of projects initiated and the outcomes for healthcare access. The final framework for the RHI can serve as a template for future academic and rural community partnerships. While there is much to learn and refine, these early steps mark a promising start toward more effective and empathetic partnerships in rural health research.

## Supporting information

Zigman Suchsland et al. supplementary material Zigman Suchsland et al. supplementary material

## Supplementary material

The supplementary material for this article can be found at https://doi.org/10.1017/cts.2024.669.
