# Supplementary material for: The rural health initiative: Bridging gaps in healthcare access and grant-funded research in central Idaho
Source: J Clin Transl Sci. 2024 Dec 12;9(1):e4. doi: 10.1017/cts.2024.669 (PMC11736294; doi:10.1017/cts.2024.669)
Supplement: Zigman Suchsland et al. supplementary material [file S2059866124006691sup001.docx]

Supplemental Material: The Rural Health Initiative: Bridging Gaps in Healthcare Access and Grant-Funded Research in Central Idaho

| S.1 Publicly Available Data Sources   1. Idaho Department of Health and Welfare 2. Idaho Coordinated Chronic Disease Plan 2014-2019 3. North Central Idaho - Community Needs Assessment 4. Institute for Health Metrics and Evaluation (IHME) United States Health Data 5. Idaho Behavioral Risk Factor Surveillance System 6. The American Communities Report 7. National Cancer Institute Cancer Statistics 8. Well Being in the Nation Network 9. Center for Disease Control and Prevention (CDC) 10. Idaho Office on Drug Policy 11. National Survey on Drug Use and Health 12. Cancer Data Registry of Idaho 13. American Cancer Society |
| --- |
